# Priming mesenchymal stem cells with α-synuclein enhances neuroprotective properties through induction of autophagy in Parkinsonian models

**DOI:** 10.1186/s13287-022-03139-w

**Published:** 2022-09-24

**Authors:** Jin Young Shin, Dong-Yeol Kim, Jieun Lee, Yu Jin Shin, Yi Seul Kim, Phil Hyu Lee

**Affiliations:** 1 Department of Neurology, College of Medicine, Yonsei University, 50 Yomsei-ro, Seodaemun-gu, Seoul, 03722 Korea; 2 Severance Biomedical Science Institute, Yonsei University College of Medicine, 50 Yomsei-ro, Seodaemun-gu, Seoul, 03722 South Korea

**Keywords:** Mesenchymal stem cells, α-Synuclein, Autophagy, Parkinson’s disease, Exosome miRNA, AMBRA1

## Abstract

**Background:**

Mesenchymal stem cells (MSCs) may be one of candidates for disease-modifying therapy in Parkinsonian diseases. As knowledge regarding the therapeutic properties of MSCs accumulates, some obstacles still remain to be overcome, especially, successful clinical translation requires the development of culture systems that mimic the natural MSC niche, while allowing clinical-scale cell expansion without compromising quality and function of the cells. In recent years, priming approaches using bioactive peptide or complement components have been investigated to enhance the therapeutic potential of MSCs.

**Methods:**

We investigated an innovative priming strategy by conditioning the MSCs with α-synuclein (α-syn). To induce priming, MSCs were treated with different concentrations of α-syn and various time course. We evaluated whether α-syn enhances stemness properties of MSCs and priming MSCs with α-syn would modulate autophagy-related gene expression profiles.

**Results:**

Treatment of naïve MSCs with α-syn upregulated transcriptional factors responsible for regulation of stemness, which was associated with the elevated expression of genes involved in glycolysis and cell re-programming. Primed MSCs with α-syn enhanced the expression of autophagy-regulating miRNA, and exosomes derived from primed MSCs were packed with autophagy-associated miRNA. In α-syn-overexpressing neuronal cells, primed MSCs with α-syn enhanced neuronal viability relative to naïve MSCs, through the induction of autophagy and lysosome activity. Animal study using an α-syn-overexpressing mice showed that the pro-survival effect of MSCs on dopaminergic neurons was more prominent in primed MSC-treated mice compared with that in naïve MSC-treated mice.

**Conclusions:**

The present data suggest that MSC priming with α-syn exerts neuroprotective effects through augmented stemness and possibly the enhancement of autophagy-mediated α-syn modulation in Parkinsonian models.

**Supplementary Information:**

The online version contains supplementary material available at 10.1186/s13287-022-03139-w.

## Introduction

Parkinson’s disease (PD) is characterized pathologically by the progressive loss of dopaminergic neurons in the substantia nigra (SN) and the presence of Lewy bodies, which are proteinaceous fibrillar cytoplasmic inclusions mainly composed of aggregated α‐synuclein (α-syn) [1, 2]. The mainstay of PD management is symptomatic treatment with dopaminergic medications [3]; however, these therapies do not affect the progressive nature of PD. Therefore, it is crucial to develop disease-modifying treatments that reduce the rate of neurodegeneration or stop the disease process in PD.

Mesenchymal stem cells (MSCs) are multipotent stem cells present in many tissues including adult bone marrow that are capable of differentiating into various cell types under appropriate conditions. MSCs exhibit neuroprotective properties via paracrine signaling, with the potential either to drive the inflammatory response toward its resolution or to strengthen it, depending on the surrounding microenvironment [4–9]. Previous studies have shown that MSCs can act as potent modulators of PD-related neurodegenerative microenvironments through the modulation of neuroinflammation and α-syn propagation, inhibition of apoptosis, increased neurogenesis and neuronal differentiation, and enhancement of autophagy [4–7, 10, 11]. However, the major challenge in MSCs-based therapies is to develop in vitro culture systems that mimic the natural MSCs niche, while allowing clinical-scale cell expansion without compromising the quality and function of the cells [12].

α-Syn is an intrinsically monomeric soluble protein and present in neuronal processes and nerve terminals [13, 14]. The level of α-syn in the cell supposedly plays a pivotal role in maintaining the balance among cell fate, cellular energy metabolism, and stress resistance in stem cell physiology [15, 16]. Interestingly, α‐syn appears to be necessary for the survival and differentiation of newly generated neurons in the dentate gyrus, but overexpression of α‐syn has a detrimental effect on the growth of neural stem cells [17, 18]. Additionally, overexpression of α-syn in mouse and human embryonic stem cells (ESCs) reduced neuro-precursor cell survival, leading to disruption of neurogenesis. ESCs-derived PD model neurons, especially those that overexpressed A53T α-syn mutation, exhibited increased susceptibility to cellular stress, including oxidative stress, proteasome inhibition, and mitochondrial inhibition, which may contribute to the pathogenesis of PD. The subcellular localization of α-syn also changes from perikarya to nerve terminal during development [19, 20], indicating that α-syn function may evolve as neural differentiation and maturation progresses. In the present study, we evaluated whether α-syn enhances stemness properties of MSCs and priming MSCs with α-syn would modulate autophagy-related gene expression profiles. Moreover, we tested whether α-syn-primed MSCs exert a neuroprotective effect in a PD animal model, thereby providing a strategy to advance MSCs application in tissue engineering.

## Materials and methods

### Cell culture

Isolation and culture of bone marrow-derived MSCs were performed as described previously. Flow cytometry analysis revealed that MSCs were positive for CD29, CD44, CD73, and CD105 and were negative for CD45 and MHC class type II (Additional file 2: Figure S2). MSCs were cultured in DMEM (Gibco BRL, Grand Island, NY, USA) supplemented with 10% FBS (Merck, Darmstadt, Germany) and 100 U penicillin–streptomycin (Gibco BRL) at 37 °C under 5% CO_2_ in a humidified incubator. SH-SY5Y cells were cultured in DMEM (Gibco BRL) supplemented with 10% FBS (Gibco BRL), 100 U penicillin–streptomycin (Gibco BRL) under identical conditions.

### α-Syn aggregate preparation and priming conditions

Recombinant α-syn (5 mg/ml in phosphate buffered saline (PBS)) was agitated at 37℃ (1000 rpm) for 5 days. An additional file shows α-syn fibrillary form to use in this study (Additional file 2: Figure S1, Additional file 1). Aggregated protein was briefly sonicated. Briefly, aggregate proteins were incubated at room temperature for 1 h. Unless otherwise stated, MSCs were seeded at a density of 5 × 10^3^ cells/cm^2^ and cultured in complete DMEM medium until 80% confluent. To induce priming, MSCs were treated with different concentrations of α-syn in DMEM for 1, 3, and 6 h.

### Cell proliferation assay

Cell viability was measured using the CellTiter 96® AQ_ueous_ One Solution Cell Proliferation Assay (Promega, Madison, WI, USA), in accordance with the manufacturer’s protocol. Briefly, after the cells were incubated with various concentrations of α-syn in DMEM, MTS (3-(4,5-dimethylthiazol-2-yl)-5-(3-carboxymethoxyphenyl)-2-(4-sulfophenyl)-2H-tetrazolium) was added to a final concentration of 0.5 mg/mL. After incubation at 37 °C for 1 h, the plates were centrifuged and the medium was aspirated from each well. Absorbance was measured at 490 nm on an ELISA microplate Versa Max reader (Molecular Devices, Sunnyvale, CA, USA).

### Quantitative real-time reverse transcription-polymerase chain reaction (qRT-PCR)

MSCs were seeded at a density of 1 × 10^5^ cells/cm^2^. Total RNA was extracted from the MSCs using TRIzol® reagent (Invitrogen, Carlsbad, CA, USA) according to the manufacturer’s protocol. An equal amount of RNA (1 µg), for each experiment, was reverse-transcribed using amfiRivert cDNA Synthesis Premix (GenDEPOT, Barker, TX, USA). Subsequently, 2 µL cDNA was used as template for qPCR with the amfiRivert 1-Step RT-PCR Kit (GenDEPOT). PCR was performed using 10 pmol primers. Primers for amplification are shown in Additional file 1: Table S1, Additional file 2. Real-time PCR was carried out with the 7300 RT-PCR system (Applied Biosystems, USA). Every assay was performed in triplicate, and all experiments included analysis of GAPDH mRNA levels as internal standard. Relative expression was determined by the Ct method, and levels were expressed as folds relative to the GAPDH mRNA levels.

### Western blot analysis

Cells and brain tissues were harvested by scraping and lysed in a buffer containing 50 mM Tris–HCl pH 8.0, 150 mM NaCl, 1% Triton X-100, 1 mM Na_3_VO4, 1% sodium deoxycholate, 0.1% SDS, 1 μg/ml pepstatin A, 50 mM NaF, 0.5 mM EDTA, 1 mM EGTA, and Protease inhibitor cocktail (Roche, Basel, Switzerland). Equal amounts of protein were resolved on an SDS-PAGE gel and transferred to a nitrocellulose membrane. The membranes were blocked by 5% skim milk for 1 h at 37 °C and then incubated with primary antibodies against BECN1-regulated autophagy protein 1(AMBRA1), microtubule-associated proteins 1A/1B light chain 3B (LC3B), α-syn, BECN, pAKT, AKT, lysosomal-associated membrane protein 1 (LAMP1), ras-related protein 7 (RAB7), transcription factor EB (TFEB) (Cell Signaling Technology, Danvers, MA, USA), and β-actin (Sigma) at 4 °C overnight. Then, the membranes were incubated with the respective secondary antibodies (GeneTex, Irvine, CA, USA) for 2 h at 37 °C. Western blot signals were visualized using the Immobilon ECL Ultra Western HRP Substrate (ECL Plus kit, GE Healthcare, Piscataway, NJ, USA) and captured on an iBright CL1000 Imaging System equipped with iBright Analysis Software (Invitrogen, Carlsbad, CA).

### Karyotype analysis

We examined the karyotype of primed MSCs and naïve MSCs in passage 8 using G-banded karyotype analysis. Briefly, metaphase chromosome spreads were prepared from cultures at the designated passages during the exponential phase of growth (65–75% confluence). For naïve MSCs and primed MSCs cultures, 0.1 μg /mL colcemid (Gibco BRL) was added directly to the cultures and incubated for two hours at 37 °C. The cells were subsequently trypsinized, fixed, and mounted on glass slides. The chromosomes were visualized by using modified Wright's staining and analyzed under a light microscope at × 10 and × 100 magnifications. Images of the individual metaphase spreads were captured and karyotyped using an automated imaging system for cytogenetics (CytoVision; Applied Imaging Corporation).

### Administration of adeno-associated virus (AAV) vector-mediated overexpression of α-syn

The plasmids for AAV vector production included the construct for the AAV2/7-serotype, the AAV viral vector transfer plasmid, and the pAdvDeltaF6 adenoviral helper plasmid. AAV serotype 2/7 expressing human wild type (WT) α-syn was driven by a human synapsin1 promoter and enhanced by a woodchuck hepatitis virus posttranscriptional regulatory element. Virus was produced by Korea Institute of Science and Technology (Seoul, South Korea). The AAV suspension is finally concentrated by iodixanol density gradient ultrafiltration and sterile filtered. The SYBR Green method was used in AAV titration to count the genome-containing particles in AAV preparations. Animals were injected with 2 µl of human WT α-syn AAV2 (~ 1 × 10^13^ genome copies per milliliter) into the right SN at a flow rate of 0.5 ml/minute.

### Animal study

All procedures were performed in accordance with the Laboratory Animals Welfare Act, the Guide for the Care and Use of Laboratory Animals, and the Guidelines and Policies for Rodent Experiments provided by the Institutional Animal Care and Use Committee at the Yonsei University Health System. Animals were acclimated in a climate-controlled room at a constant 12 h light/dark cycle for 1 week prior to the initiation of the drug administration. To evaluate the effects of MSCs and primed MSCs on α-syn modulation, the mice were randomly divided into the following four groups (*n* = 6 per group): (1) vector-only (control); (2) AAV-WT α-syn; (3) AAV-WT α-syn with MSCs (naïve MSC group) and (4) AAV-WT α-syn with primed MSCs (primed MSC group). Mice were injected with naïve MSCs (1 × 10^7^cells/kg) or primed MSCs (1 × 10^7^cells/kg) via tail vein on postoperative day 7. All mice were killed 1 month postoperative. All mice were killed 1 month postoperative.

### Brain sample preparation

For immunochemical analysis, all mice were deeply anesthetized with chloral hydrate (intraperitoneal injection, 0.4 g/kg; Sigma) and then, perfused with 4% paraformaldehyde (Sigma) in 0.1 M phosphate buffer (pH 7.4). The brains were harvested from the skulls, post-fixed overnight in 4% paraformaldehyde, and stored in 30% sucrose solution for 1–2 days at 4℃ until they sank. Finally, 25-μm coronal sections were obtained using cryostat. The sections were stored in tissue storage solution (30% glycerol, 30% ethylene glycol, 30% distilled water, 10% 0.2 M PB) at 4℃ until required.

### Immunohistochemistry

Brain sections were washed twice in PBS and incubated in 0.5% Triton X-100 (Sigma) for 15 min at room temperature. They were blocked with 5% bovine serum albumin (BSA; Sigma) for 30 min. After blocking, they were incubated overnight at 4 °C with specific primary antibodies. The primary antibody was used mouse anti-tyrosine hydroxylase (TH) (Sigma, T2928). The TH antibodies were detected using 0.05% diaminobenzidine (DAB, Vector Laboratories, USA). The immune-stained cells were analyzed using bright-field microscopy and viewed under a Zeiss LSM 700 confocal imaging system (Zeiss, Germany).

### Glucose uptake measurement

The assay was performed essentially as described previously (Perrini et al. 2004). The cells were washed thrice with PBS, then incubated in Krebs–Ringer phosphate buffer (KRP, 1.32 mM NaCl, 4.71 mM KCl2, 47 mM CaCl2, 1.24 mM MgSO4, 2.48 mM Na3PO4, 10 mM HEPES (pH 7.4)) for 10 min at 37 8C, then 0.5 mCi/ml 2-DOG as the final concentration was added to the cells. After 10-min incubation, the medium was aspirated and the cells were washed thrice with ice-cold KRP containing 10 mM glucose to terminate the reaction. The cells were lysed with 0.1 M NaOH, and the radioactivity taken up by the cells was determined using a scintillation counter (Beckman Instruments, Fullerton, CA, USA). The d.p.m. value was corrected by protein content in each well which was measured using a BCA protein assay kit.

### L-lactate assay

Intracellular lactate levels were measured using a colorimetric L-lactate assay kit (Abcam, ab65330), according to the manufacturer’s instructions. Cell lysates were deproteinized to eliminate the endogenous lactate dehydrogenases (LDH) and then, incubated in the presence of the lactate probe and enzyme mix at room temperature for 30 min. Absorbance was measured at 570 nm using a microplate reader.

### Data and statistical analysis

The data and statistical analysis comply with the recommendations of the British Journal of Pharmacology on experimental design and analysis in pharmacology [21]. The group means were compared using the Mann–Whitney U-test for pairs and the Kruskal–Wallis analysis for multiple groups. *P* values less than 0.05 were considered statistically significant. Statistical analyses were performed using the commercially available software SPSS (version 12.0).

## Results

### MSCs primed with α-syn show heterogeneity in their proliferation rate and senescence status

**Fig. 1:**
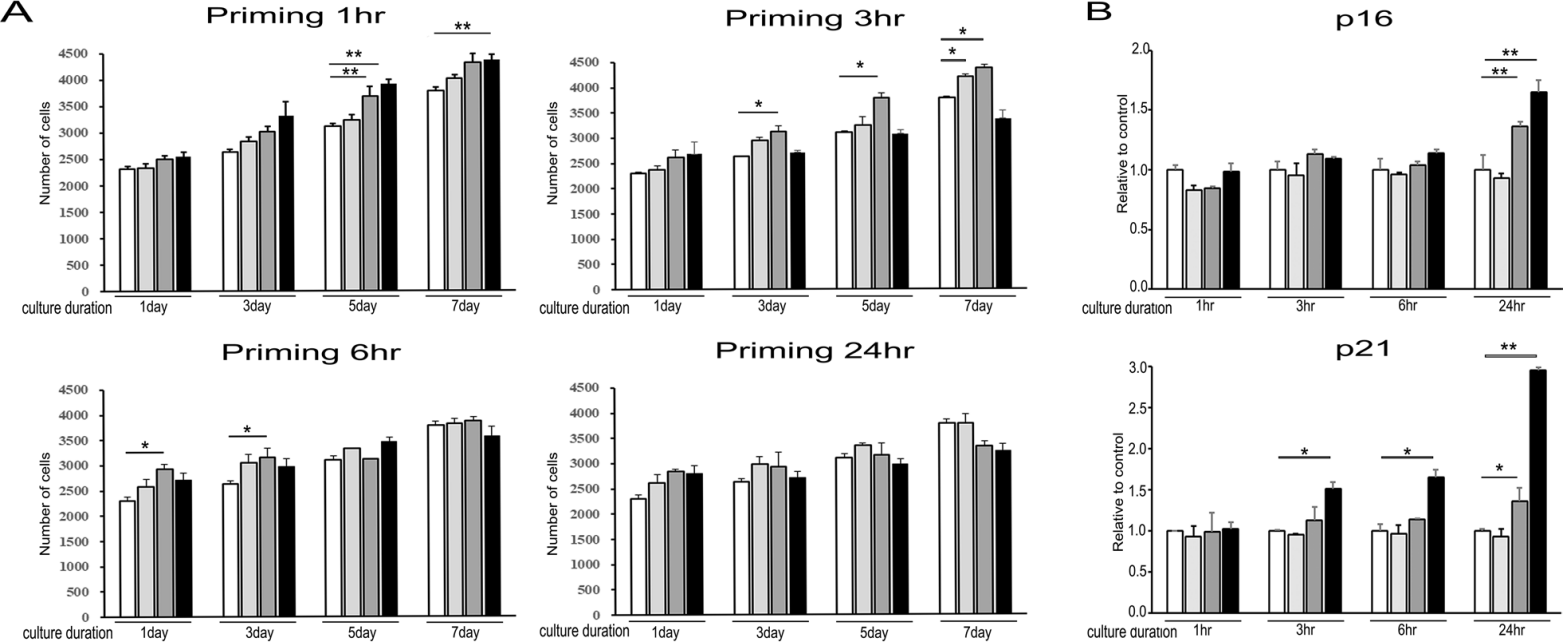
α-Syn enhances MSC proliferation and does not alter senescence status. **A** Cell proliferation assay showed a significant increase in cell density of α-syn-treated MSCs compared to control MSCs in a time-dependent manner, regardless of α-syn concentration. **B** Quantitative RT-PCR shows that the expression levels of the senescence markers *p16* and *p21* do not increase after incubating for 6 h with various α-syn concentrations but increases after 24 h

α-Syn-primed MSCs displayed a significant increase in cell density compared with that of control MSCs in a time and dose-dependent manner. MSCs exhibited a higher proliferation rate when treated with 0.5 and 1 µM α-syn for 1 h and cultured for 5 days. Additionally, a higher proliferation rate was observed on treatment with 0.5 µM α-syn for 3 h followed by culturing for 3 days, and 0.1 µM for 3 h followed by culturing for 7 days. While a higher proliferation rate was initially observed in MSCs primed with 1.0 µM α-syn for 6 h (after 1 and 3 days culture), the rate reduced on culturing for 5 days (Fig. 1A). Longer exposure times for α-syn treatment did not result in any significant increase in MSC cellular density compared to that of naïve MSCs regardless of the α-syn concentration and culture duration. We then analyzed the expression of senescence-related genes in primed MSCs to evaluate their senescence status. The expression levels of both p16 and p21 increased on treatment with 0.5 and 1 µM α-syn for 24 h, but were not affected by a lower α-syn concentration (0.1 µM). p21 was especially more sensitive to α-syn treatment at 1 µM for 3 h and 6 h (Fig. 1B). Karyotype analysis showed that primed MSCs had a normal karyotype with a diploid chromosome number as naïve MSCs (Additional file 2: Figure S2, Additional file 1). Taken together, these results suggest that α-syn plays a role in MSC proliferation under priming conditions.

### Priming MSCs with α-syn boosts their stemness capacity but does not alter expression of differentiation-related genes

**Fig. 2:**
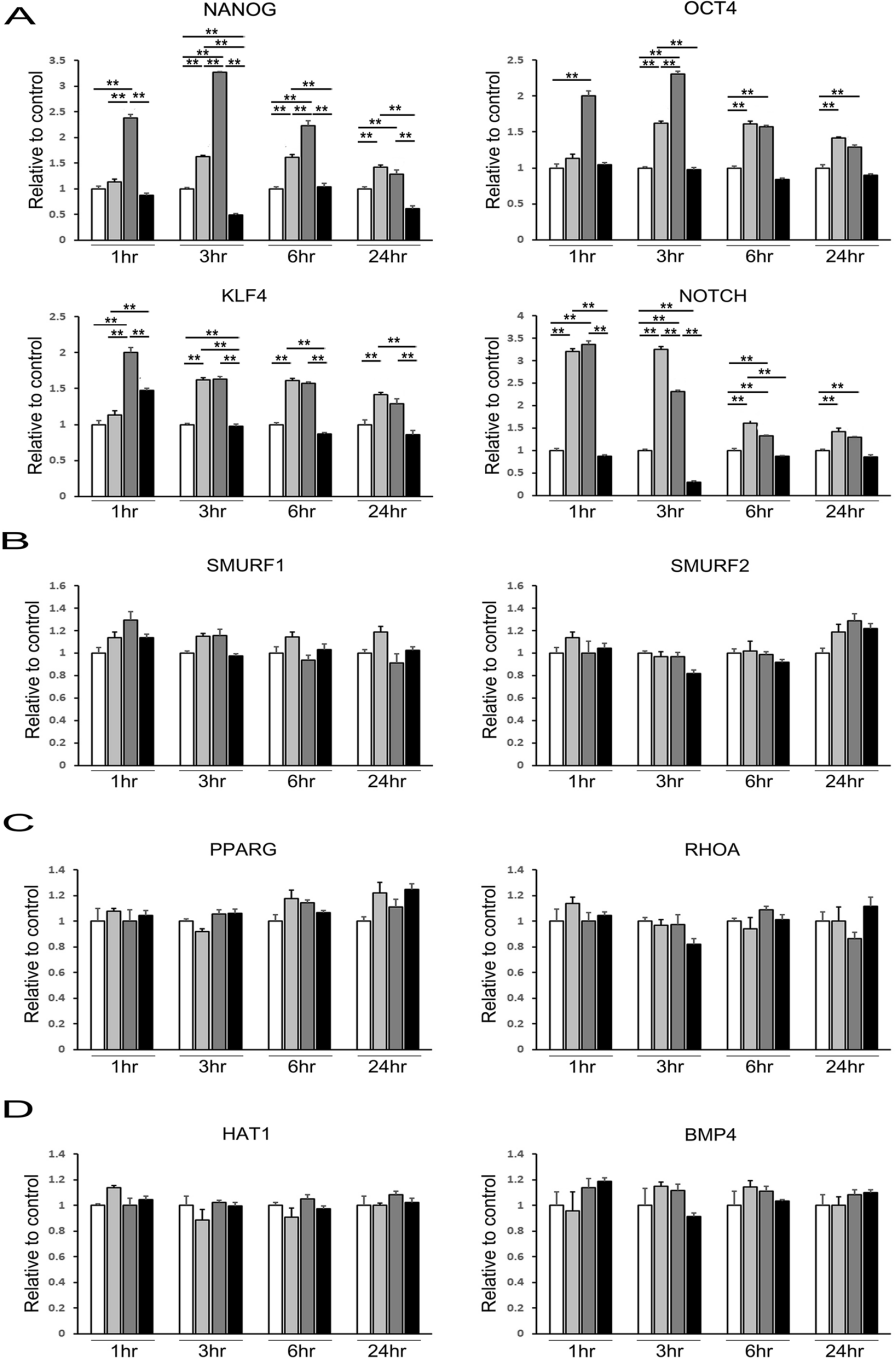
α-Syn regulates MSC stemness and differentiation potential. **A** Expression levels (determined by quantitative RT-PCR) of the stemness markers *OCT4*, *NANOG*, *SOX2,* and *NOTCH* increase after 24 h incubation with 0.1 μM and 0.5 μM α-syn, but decrease on treatment with higher α-syn concentration. **B**–**D** Quantitative RT-PCR shows that α-syn treatment does not affect the expression levels of differentiation markers for **B** osteogenesis (*SMURF1, SMURF2*), **C** adipogenesis (*PPARG, RHOA),* and **D** chondrogenesis (*HAT1, BMP4*)

Given that multipotent differentiation is a major characteristic of MSCs, we investigated the effects of α-syn on the stemness capacity and expression of differentiation-related genes in primed MSCs. Treatment with α-syn at 0.1 µM and 0.5 µM increased the mRNA levels of transcriptional factors responsible for stemness regulation, such as homeobox protein NANOG (NANOG), octamer-binding transcription factor 4 (OCT4), kruppel-like factor 4 (KLF4), and neurogenic locus notch homolog protein (Notch) in a dose and time-dependent manner. However, priming MSCs with 1 µM α-syn caused a significant reduction in the expression of the transcriptional factors relative to priming conditions with low α-syn concentrations in a time-dependent manner (Fig. 2A). To test whether α-syn-induced stemness augmentation would additionally enhance the differentiation potential of MSCs, we examined the mRNA levels of genes associated with differentiation, including osteogenesis-related protein, E3 ubiquitin-protein ligase SMURF1,2 (SMURF1, SMURF2), adipogenesis-related protein, peroxisome proliferator-activated receptor gamma (PPARG), transforming protein RHOA (RHOA), and chondrogenesis-related protein, histone acetyltransferase 1 (HAT1), bone morphogenetic proteins 4 (BMP4). Priming MSCs with α-syn did not change the expression levels of osteogenesis-, adipogenesis-, and chondrogenesis-related genes in a time-dependent manner, regardless of α-syn concentration (Fig. 2B–D).

## MSC priming with α-syn increases glycolysis by upregulating PFKFB3 and PKM

**Fig. 3:**
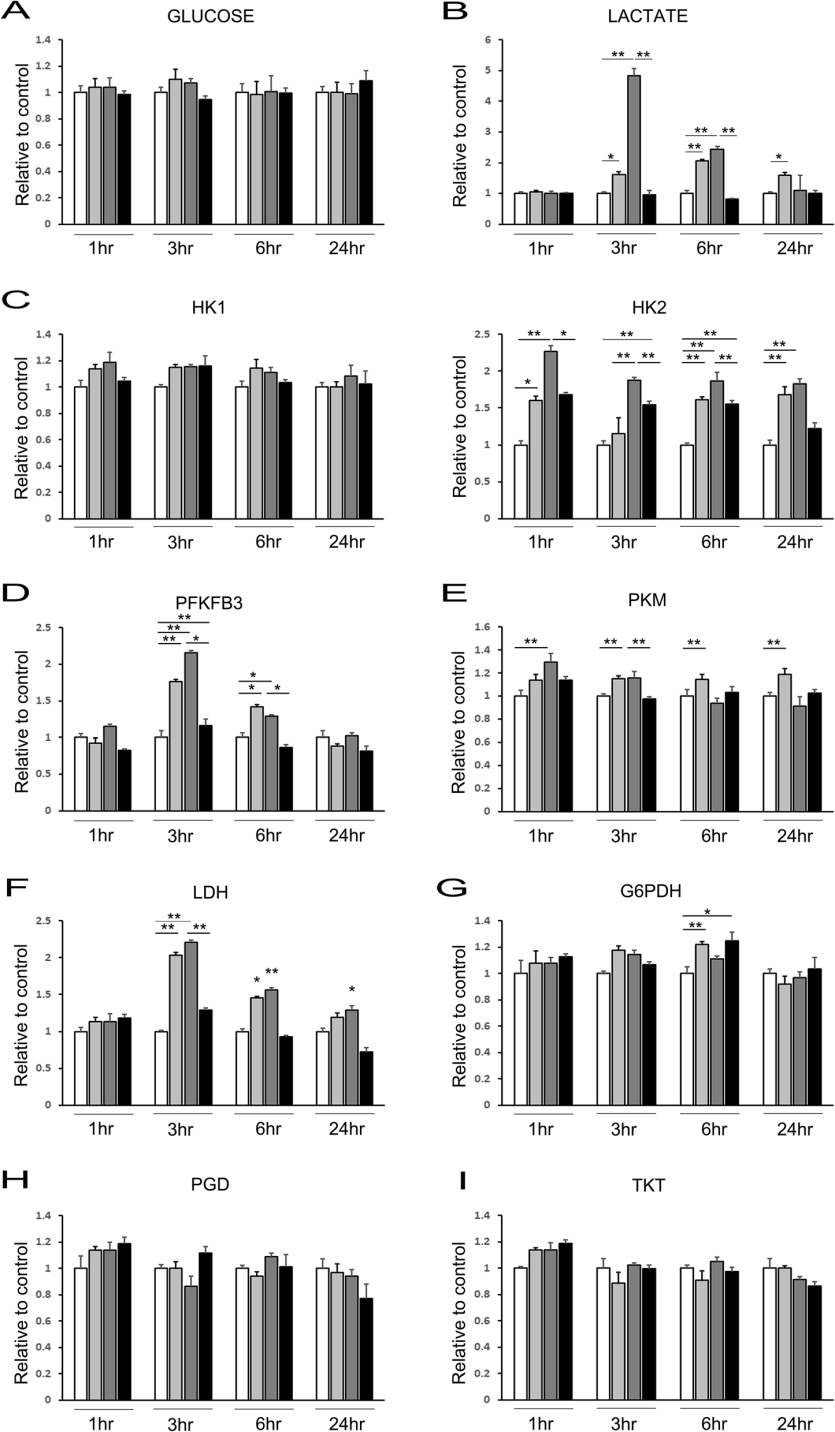
α-Syn enhances MSC glycolysis by upregulating PKM and LDH. **A** Glucose uptake by MSC is not affected by α-syn-treatment. **B** α-Syn-treated MSCs present an increased accumulation of lactate compared with that by control MSCs. **C**–**F** α-Syn-treated MSCs exhibit increased mRNA levels of glycolysis enzymes, such as HK1, HK2, PFKFB3, PKM, and LDH, compared to the control MSCs. **G**–**I** mRNA levels of pentose-phosphate pathway enzymes, such as G6PD, PGD, and TKT, show no significant differences in α-syn-treated MSCs compared with that of control MSCs. Data are presented as mean ± SE. *P < 0.05; **P < 0.01

To demonstrate whether MSC priming with α-syn relies on glycolysis, we analyzed the expression levels of the genes encoding the rate-limiting enzymes in the pathway by quantitative RT-PCR. Primed MSCs showed slightly higher glucose uptake than naïve MSCs in all priming conditions, but produced a 1.6 to 4.8-fold higher concentration of lactate in primed MSCs on 3 h priming with α-syn (Fig. 3A, B). Hexokinase 1/2 (HK1/2) and 6-phosphofructo-2-kinase/fructose-2,6-bisphosphatase 3 (PFKFB3) function in the first two irreversible steps of glycolysis. Notably, HK2 and PFKFB3 displayed higher expression in all priming conditions with the exception of 0.1 µM α-syn for 3 h, while that of HK1 (Fig. 3C, D). The expression of a majority of glycolysis genes, pyruvate Kinase M (PKM) was increased in primed MSCs; particularly, the expression of genes that encode enzymes from PFKFB3 to LDHA displayed up to twofold increase in expression compared with that of naïve MSCs (Fig. 3E, F). In contrast to the significant increment in the expression of glycolysis genes, the levels of several pentose-phosphate pathway genes remained unaltered in primed MSCs. Only the expression of glucose-6-phosphate dehydrogenase (G6PDH), phosphogluconate dehydrogenase (PGD), and transketolase (TKT) increased by nearly 20% (Fig. 3G–I). These results suggest a conversion from aerobic respiration to glycolytic metabolism in primed MSCs.

### Priming MSCs by α-syn enhances autophagy- and stemness-regulating miRNA expression

**Fig. 4:**
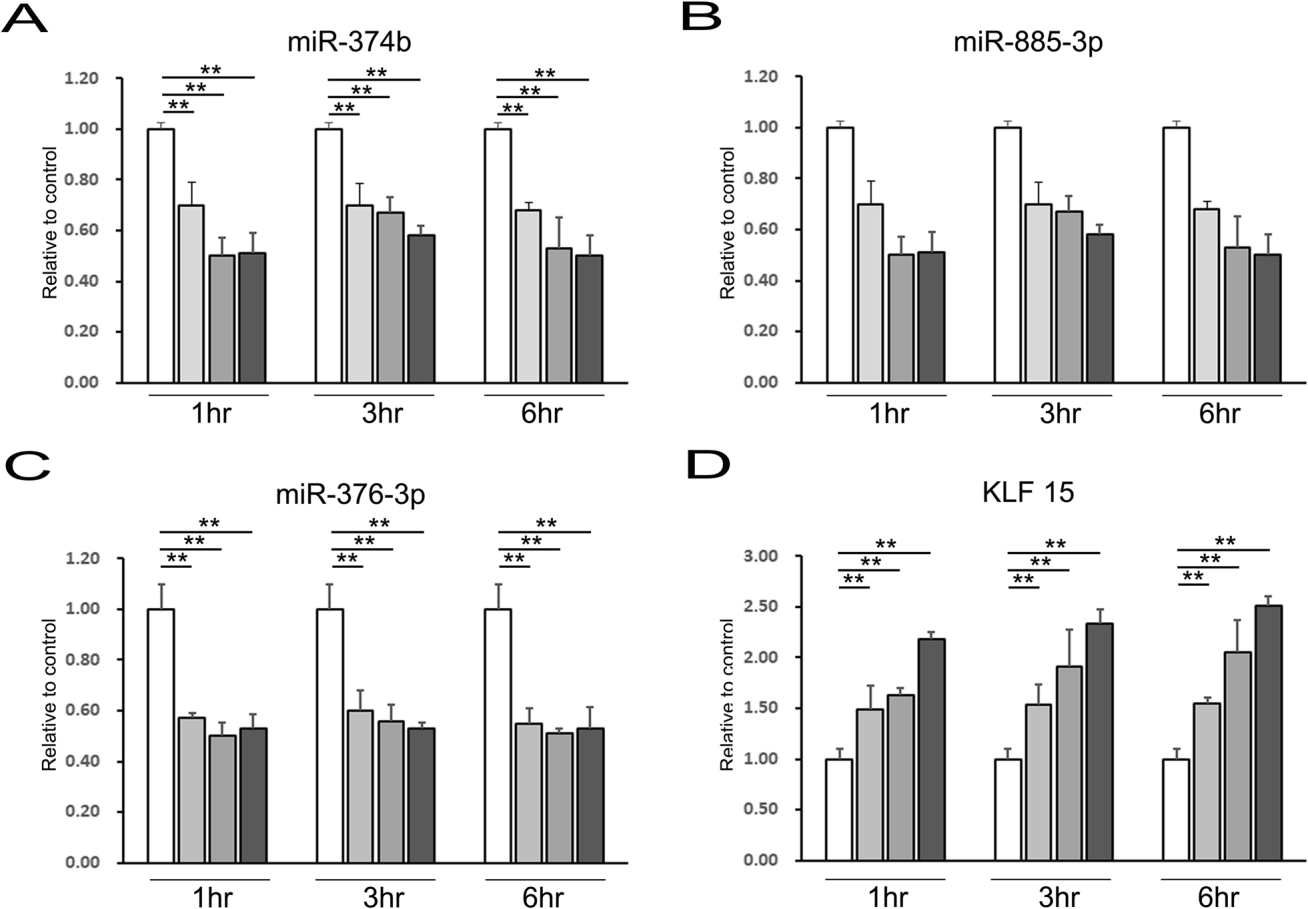
miR-374b and miR 376-3p regulate stemness of α-syn-associated MSC priming for targeting *KLF15*. **A**–**C** Quantitative RT-PCR for *KLF15*-related miRNA shows a decrease in miR-374b and miR-376-3p expression levels but no significant change in that of miR-885-3p in α-syn-treated MSCs compared to those in control MSCs. **D** Quantitative RT-PCR analysis shows that downregulation of *KLF15*-related miRNA leads to increased mRNA levels of *KLF15*. Data are presented as mean ± SE. *P < 0.05; **P < 0.01

Next, we examined the expression of autophagy-related miR-374b (induction), 885-3p (vesicle nucleation), and 376b-3p (vesicle elongation) in primed MSCs. In the absence of priming condition, autophagy-related miRNA had no significant effect in primed MSCs. However, the expression of miR374b and 376b was significantly attenuated in primed MSCs than that in naïve MSCs. Notably, expression of 376-3p was more significantly decreased compared to that of miR-374b (Fig. 4A, C). The expression of miR885-3p also tended to be lower in primed MSCs (Fig. 4B). This result suggests miR 376-3p as a candidate regulator of α-syn-associated MSC priming. We further analyzed miRNA-target protein prediction databases (http://www.targetscan.org) to screen miR376-3p-targeting stemness-related genes. The TargetScan database predicted 258 broadly conserved miRNA families, of which miR376-3p was found to target the stemness-related gene kruppel Like Factor 15 (KLF15) (Fig. 4D). The expression of KLF15 was increased in MSCs primed with 0.1, 0.5, and 1 µM α-syn for 6 h (Additional file 2: Fig. S1).

### Exosomes derived from primed MSCs are packed with autophagy-associated miRNA

**Fig. 5:**
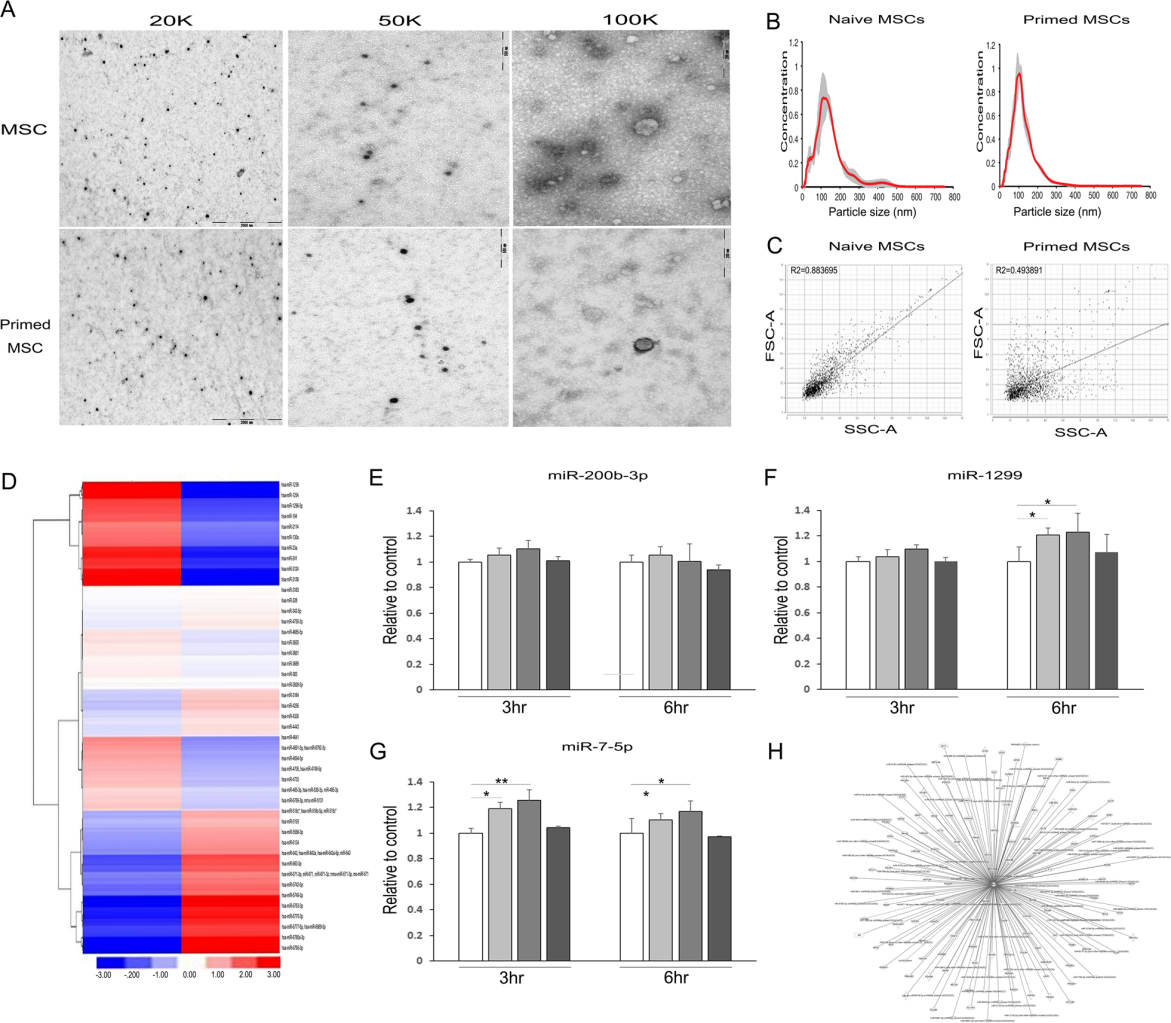
Exosomes derived from primed MSCs are packed with autophagy-associated miRNA. **A** MSC and primed MSC-derived exosomes, diameter 50–500 nm, as visualized by EM and PTA staining at 20 K × , 50 K × , and 5 K × magnifications. Scale bar indicates the size of the field shown. **B**, **C** Characterization of exosomes using NTA and SP-IRIS immunophenotypic analysis. NTA was performed to measure the total number of particles (**B**) and particle distribution (**C**). **D** Comparison of miRNA in MSCs and primed MSC-derived exosomes. Heatmap shows unsupervised hierarchical clustering of samples, generated with Multi-Experiment Viewer (MeV v4.9). **E**–**G** Quantitative RT-PCR analysis for autophagy-related miRNA shows that miR-200-3pb, miR1299, and miR-7-5p expression levels increase in α-syn-treated MSCs compared to those in control MSCs, but the change in miR-200b-3 pp expression is not significant. **H** Network analysis with IPA shows that miR-7-5p miR-1299a, and miR-200b-3p targeted genes are involved in AMBRA1-induced autophagy cascade

We sought to determine how the priming of MSCs exposed to α-syn for 3 h and 6 h was associated with their exosome and exosomal miRNA profiles. Using nanoparticle tracking analysis (NTA) and transmission electron microscopy (TEM), we observed that particles were present in the size of extracellular vesicles (between 50 and 500 nm) with enrichment in small particles (mode size 137 ± 13 nm) within exosomes (Fig. 5A, B). To analyze the changes in the expression of primed MSC-derived exosomal miRNA, we detected the global expression profiles of exosomal miRNA in primed MSCs and naïve MSCs and used the R package to screen the differential expression of miRNAs following the criteria: *q* value ≤ 0.05; fold change ≥ 2 or ≤ 0.5. We could intuitively observe this phenotype in a log–log scatter plot (Fig. 5C). We further used microarray profiling of miRNAs in primed and naïve MSCs to identify the factors and the mechanism responsible for the beneficial effects of primed MSCs derived exosomal miRNA. We examined the exosomal miRNA content by using microarray profiling of the miRNAs in primed MSCs and naïve MSCs because exosomal proteins or mRNAs/miRNAs that mediate their biological effect. Specifically, we focused on the exosomal miRNAs upregulated in primed MSCs in comparison with naïve MSCs. Using ANOVA to screen the differential expression of miRNAs at *P*-value ≤ 0.01, fold change ≥ 2 or ≤ 0.5, 666 miRNAs were detected (Fig. 5D). To further filter the key miRNA in primed MSCs, we defined differential miRNAs using the criteria: q value ≥ 0.05; fold change ≥ 5 or ≤ 0.2. Three miRNAs (miR-7-5p, miR-1299a, and miR-200b-3p) were found to exhibit specific expression in primed MSC-derived exosomes. Based on the miRNA profiling data from microarray sequencing, we further validated the expression of these miRNAs by qRT-PCR (Fig. 5E–G). Intriguingly, network analysis with Ingenuity Pathway Analysis (IPA) showed that miR-7-5p, miR-1299a, and miR-200b-3p targeted genes involved in the AMBRA1-induced autophagy cascade (Fig. 5H).

### Primed MSCs enhance neuroprotective properties through induction of autophagy

**Fig. 6:**
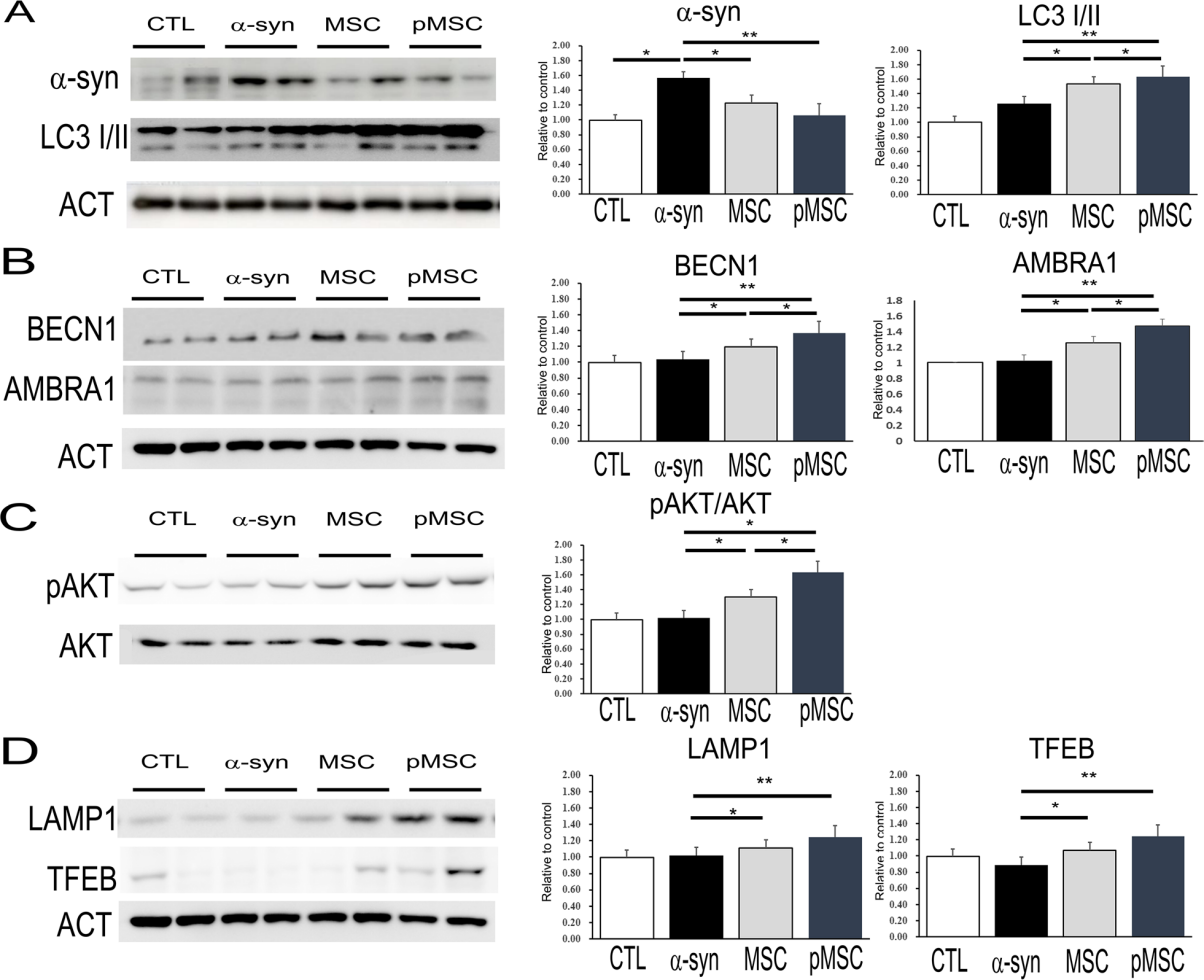
Primed MSCs enhance neuroprotective properties through induction of autophagy in α-syn-overexpressing SH-SY5Y cells. **A**, **B** Western blotting for autophagy marker shows that co-culture with primed MSCs significantly reduced α-syn and enhances LC-3, Beclin-1, and AMBRA1 expression compared to the co-culture with control or naïve MSCs. **C** Western blotting shows that co-culture with primed MSCs significantly enhances the expression of autophagy flux-related markers, p-Akt and Akt, and **D** lysosome activity markers, TFEB, compared to the α-syn-overexpressing control or co-culture with naïve MSCs. The levels of target protein were quantified and normalized to β-actin using ImageJ. Data are presented as mean ± SE. *P < 0.05; **P < 0.01

**Fig. 7:**
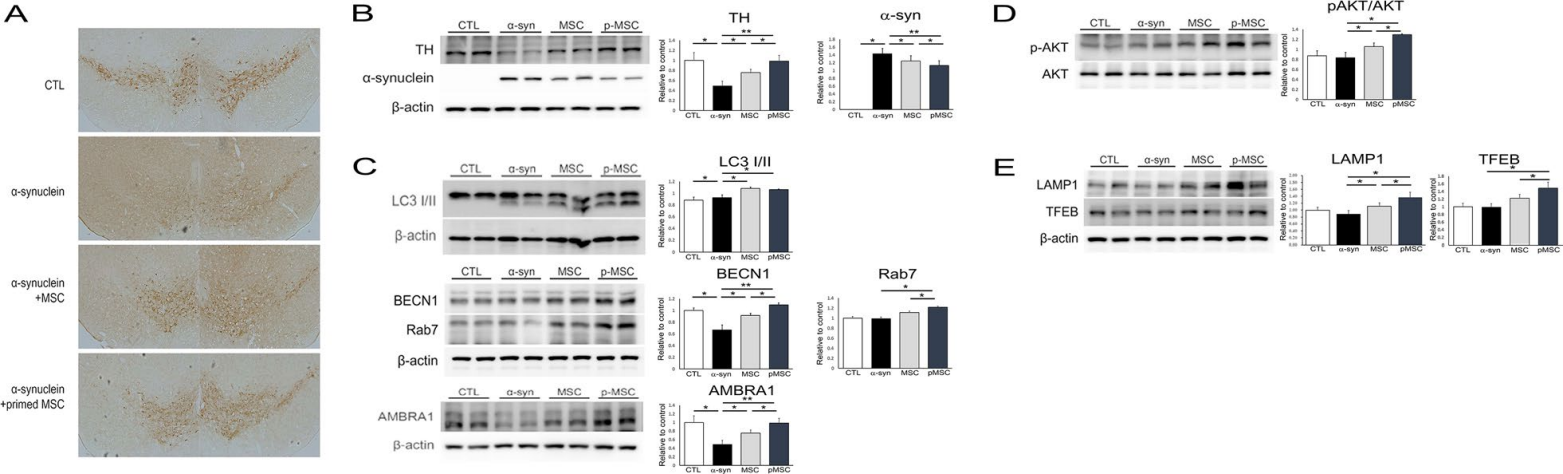
MSC priming exerts neuroprotection in α-syn-overexpressing mice. **A** TH-positive neuron counting in the SN at 4 weeks reveals that naïve and primed MSC treatment induces higher numbers of dopaminergic neurons compared to the α-syn-overexpressing control MSC group. **B** Western blotting shows that both naïve and primed MSC treatment significantly increases TH expression levels compared to the α-syn-overexpressing control MSC group. **C** Western blotting for autophagy markers shows that naïve and primed MSC treatments significantly upregulate autophagy-related markers, LC-3, Beclin-1, and AMBRA1 compared to the α-syn-overexpressing control MSC group. **D** Both naïve and primed MSC treatments lead to an increase in the expression of autophagy flux-related marker p-Akt/Akt and **E** lysosome activity markers, LAMP 1 and TFEB, compared to the control MSC group. The levels of target protein were quantified and normalized to β-actin using ImageJ. Data are presented as mean ± SE. *P < 0.05; **P < 0.01

To identify whether primed MSCs increase the level of autophagy in α-syn-overexpressing SH-SY5Y cells (Parkinsonian cellular model), the MSCs were incubated in co-culture (no cell-to-cell contact) for 24 h and then, examined by Western blotting. Immunoblotting revealed that the α-syn level in SH-SY5Y cells tended to be lower when they were co-cultured with primed MSCs relative to naïve MSCs, and the ratio of LC3B-II/LC3B-I tended to be higher in the primed MSC group (Fig. 6A). Next, we examined autophagy activity in the α-syn-overexpressing SH-SY5Y cells that were co-cultured with primed MSCs. The expression of AMBRA1 and BECN1 was significantly higher in neuronal cell co-cultured with primed MSCs relative to naïve MSCs (Fig. 6B). Therefore, these results suggest that primed MSCs have a great capacity for regulating autophagy in SH-SY5Y cells. As autophagosome formation requires class III PI3-kinase activity, the activation of AKT was examined by Western blotting using phosphorylated antibodies. Co-culture with primed MSCs increased the levels of phosphorylated AKT (pAKT) in α-syn-overexpressing SH-SY5Y cells compared to that when co-cultured with naïve MSCs (Fig. 6C). Finally, we examined whether the primed MSCs-induced autophagy flux via lysosome activity. The expression of LAMP-1 and TFEB tended to be higher in the primed MSC group relative to the naïve MSC group (Fig. 6D). Furthermore, we examined whether primed MSCs could enhance autophagy-mediated neuroprotective effect in Parkinsonian animal using AAV vector-mediated overexpression of α-syn. Both naïve and primed MSCs increased the survival of TH-positive neurons in the SN compared to α-syn-overexpressing mice. However, MSC priming yielded higher numbers of TH-positive neurons in the SN compared to mice treated with naïve MSCs (Fig. 7A, B). In addition, mice treated with primed MSCs exhibited lower α-syn expression in the midbrain compared to the mice receiving naïve MSCs (Fig. 7B). Administration of primed MSCs also significantly increased the expression of autophagy initiation-related proteins, including LC3, BECN1, RAB 7 and AMBRA1 (Fig. 7C). Finally, we investigated whether primed MSCs-induced autophagy occurred through central regulator in AKT-modulated autophagy induction. The levels of pAKT increased in mice treated with primed MSCs compared to those treated with naïve MSCs (Fig. 7D). As expected, this change was also accompanied by a significant increase in lysosome activity in primed MSC-treated mice compared to naïve MSC-treated mice (Fig. 7E).

## Discussion

In the present study, we explored whether priming MSCs with α-syn would enhance stemness and neuroprotective properties in Parkinsonian models. The major findings are as follows: (1) α-syn treatment in naïve MSCs upregulated transcriptional factors responsible for regulation of stemness, which was associated with an increase in glycolysis and cell priming, (2) priming MSCs with α-syn enhanced autophagy-regulating miRNA expression, and exosomes derived from primed MSCs were packed with autophagy-associated miRNA, (3) priming MSCs with α-syn enhanced neuronal viability in the Parkinsonian cellular model, relative to naïve MSCs, through the induction of autophagy and lysosome activity, and (4) in α-syn-overexpressing Parkinsonian animal model, the pro-survival effect of MSCs on dopaminergic neurons was more prominent in primed MSC-treated mice compared to that in naïve MSC-treated mice. These data suggest that MSC priming with α-syn can provide a strategy to improve the application of MSCs in treating Parkinsonian disorders.

Many studies have tried to identify the ‘‘stem cell niche,’’ consisting of various microenvironmental factors that regulate the balance of self-renewal and differentiation [22]. Several cellular and non-cellular factors have been defined, such as BMP, WNT, Notch signaling, matrix glycoproteins, blood vessels, and the three-dimensional space [23]. Hypoxia has been proposed as one of the microenvironmental factors that controls self-renewal and enhances specific stem cell qualities of a variety of stem cells, including NSCs, ESCs, HSCs [24–26]. Recently, uric acid could provide novel strategy to improve the application of MSCs in Parkinsonian disorders. The data suggest that MSC priming with uric acid exerts neuroprotective effects through enhanced stemness and differentiation potential in Parkinsonian models [27]. In this study, we demonstrated that MSCs can steadily exhibit self-renewal under α-syn treatment conditions. The properties of primed stem cells include upregulation of stemness-associated transcription factors and downregulation of lineage differentiation-specific markers. Stemness-associated transcription factors such as OCT4, NANOG, and KLF4 are considered to form a transcriptional regulatory circuit for pluripotency and self-renewal of stem cells, including MSCs [28, 29]. NANOG and OCT4 support the expression of each other and other self-renewal genes and repress the expression of differentiation-related genes [30]. KLF4 induces the expression of not only NANOG but also other oncogenes, which promote proliferation unrelated to the function of NANOG in priming MSCs [31, 32]. In this context, our results showed that α-syn treatment in MSCs increases the expression of self-renewal markers (e.g., OCT4, NANOG, and KLF4) but does not affect the expression of lineage differentiation markers (e.g., SMURF1/2, PPARG, and BMP4).

Another crucial property of stem cells is the ability to modulate their metabolism. Cellular metabolism is not a passive player in the process of stem cell lineage commitment; rather, many important cell fates are regulated through metabolic changes [33]. This generally implies shifting from glycolysis to oxidative phosphorylation (OXPHOS), when moving from an undifferentiated to a differentiated state [34]. Interestingly, naïve and primed pluripotent stem cells (PSCs) exhibit widely different metabolic signatures. Primed PSCs are unusual in that they generate most of their ATP from glycolysis rather than the more familiar mode of energy generation through OXPHOS as observed in naïve PSCs [35]. In the present study, we found that MSCs primed with α -syn exhibit an altered glucose metabolism, slightly increasing their glucose uptake and producing significantly more lactate than their naïve counterparts. Furthermore, priming MSCs with α-syn stimulated glycolysis with no changes to OXPHOS. Specifically, glycolysis-related enzymes such as PFKFB3, PKM, and LDH were significantly upregulated, which was accompanied by a marked increase in the rate of cell proliferation. In addition, low-concentration and short-incubation α-syn treatment did not affect the expression of OXPHOS-related genes, such as G6PDH, PGD, and TKT, indicating no effect on the differentiation potential of MSCs. Accordingly, our results suggest that α-syn enhances MSC stemness, with strict regulation of glycolysis, possibly dependent upon α-syn concentration and incubation time.

The production of secretory autocrine and paracrine factors that play critical roles in tissue repair augments the potential of MSCs in regenerative medicine. Therefore, modulation of the MSC-derived secretome is an important strategy for improving the therapeutic effect of MSCs [36–38]. Here, we found that among the known miRNAs, miR 376-3p and miR-374b would act as a candidate regulator of stemness in primed MSCs by α-syn via counteracting stemness-related KLF15. There are three members in miR-376 family: miR-376a, miR-376b, and miR-376c. Several studies have demonstrated that miR-376b is involved in many biological processes, such as autophagy, red blood cell differentiation, and decision of cell phenotype [39] and can regulate cell proliferation and apoptosis via targeting on KLF15 [40, 41]. In addition, miR-374b can affect PKM gene splicing, which promotes aerobic glycolysis and stemness by high levels of PKM2 [42, 43]. Thus, it is plausible that α-syn-induced miR-374b downregulation and subsequently elevated glycolysis seem to result in increased MSC stemness primed by α-syn. Moreover, network analysis with IPA revealed that exosomes derived from α-syn-primed MSCs were packed with autophagy-associated miRNA, including miR-7-5p, miR-1299a, and miR-200b-3p. Among these miRNAs, we found that miR-7-5p was also upregulated in α-syn-overexpressing neuronal cells co-cultured with primed MSCs. We consider that the upregulation of intracellular miR-7-5p in α-syn-overexpressing neuronal cells may be ascribed to the uptake of exosomes derived from primed MSCs. Although miR-7-5p is known to be an important regulator in autophagy of various cell types [44–46], there are no studies exploring the relationship between miR-7-5p and AMBRA1. Our data revealed that miR-7-5p from primed MSCs induced autophagy in α-syn-overexpressing neuronal cells depending on BECN1 and another key endpoint of AMBRA1. Specifically, we found that upregulation of Akt phosphorylation was associated with reversing the effect of α-syn-primed MSCs by upregulating miR-7-5p and consequently increased AMBRA1 level in α-syn-overexpressing neuronal cells. Thus, the indirect interaction between AMBRA1 and miR-7-5p may play an important role in autophagy induction in α-syn-overexpressing neuronal cells co-cultured with α-syn-primed MSCs.

Autophagy is induced by various stimuli including nutrient deprivation, misfolded or aggregated proteins, or damaged organelles to maintain intracellular homeostasis [47, 48], and dysregulation of autophagy is a key player in the pathogenesis of PD [49]. In the present study, we demonstrated that priming MSCs with α-syn resulted in beneficial neuroprotective effects in α-syn-overexpressing Parkinsonian cellular and animal models through possibly enhanced autophagy-mediated α-syn modulation. In the cellular PD model, primed MSCs tended to have a high capacity for the clearance of α-syn with increased autophagy and lysosome relative to naïve MSCs. Similarly, animal study showed that priming MSC with α-syn yielded higher numbers of dopaminergic neurons in the SN compared to naïve MSCs-treated mice, which was accompanied by decreased expression levels of α-syn and an increase in Akt-modulated autophagy induction and lysosome activity.

## Conclusions

**Fig. 8:**
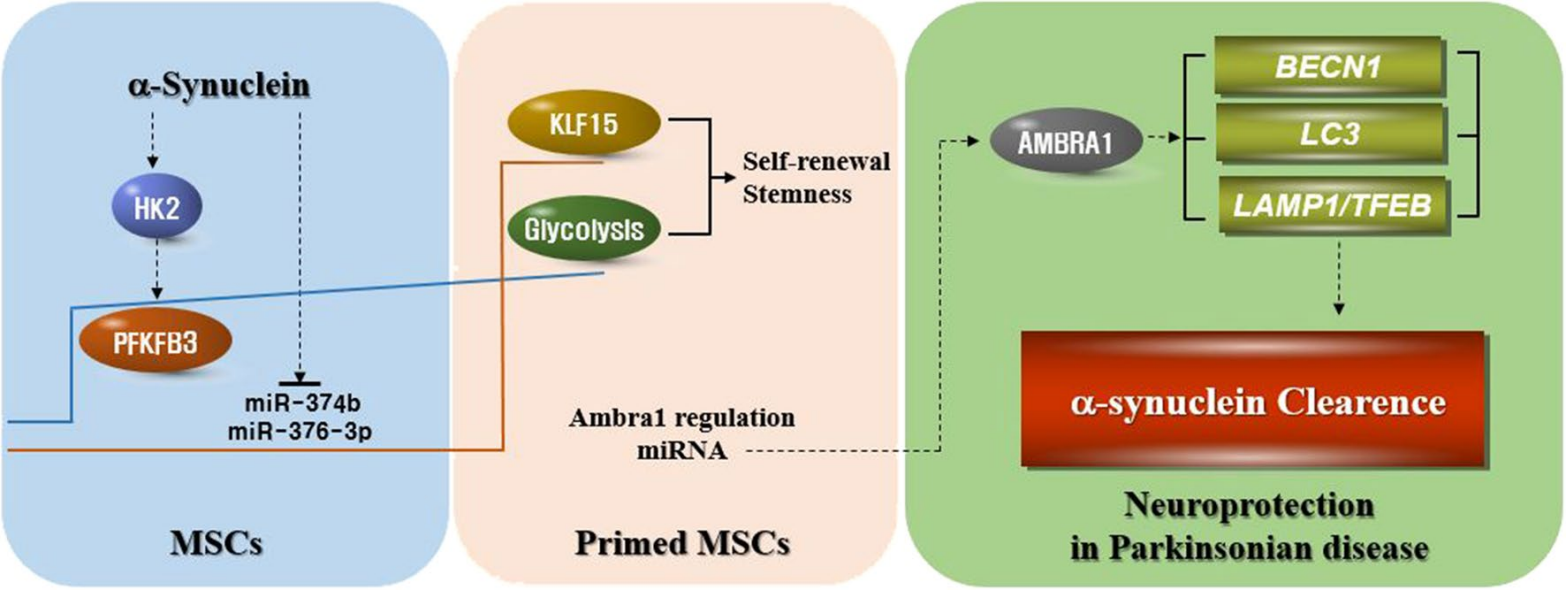
MSC priming with α-syn exerts neuroprotective effects through augmented stemness and the enhancement of autophagy-mediated α-syn modulation in parkinsonian models

The present data suggest that MSC priming with α-syn exerts neuroprotective effects through augmented stemness and possibly the enhancement of autophagy-mediated α-syn modulation in Parkinsonian models (Fig. 8). Therefore, priming could provide a novel strategy to improve MSC application for the treatment of Parkinsonian disorders.

## Supplementary Information

**Additional file 1**.** S1 table**. RT-qPCR prime sequence. Forward and reverse primer sequences were designed using Prime-BLAST.

**Additional file 2**.** Fig. S1**. A characterization of fibrillary form of α-synuclein using the transmission electron microscopy.** Fig. S2**. A karyotype analysis of primed MSCs. Primed MSCs with α-synuclein had a normal karyotype with a diploid chromosome number as naïve MSCs in passage 8.

## Data Availability

Data and materials will be provided upon private request.

## Abbreviations

PD: Parkinson’s disease
SN: Substantia Nigra
α-syn: α‐Synuclein
MSCs: Mesenchymal stem cells
ESCs: Embryonic stem cells
AAV: Adeno-associated virus
TH: Tyrosine hydroxylase
LDH: Lactate dehydrogenases
NANOG: Homeobox protein NANOG
OCT4: Octamer-binding transcription factor 4
KLF4: Kruppel-like factor 4
Notch: Neurogenic locus notch homolog protein
SMURF1, SMURF2: E3 ubiquitin-protein ligase SMURF1,2
PPARG: Peroxisome proliferator activated receptor gamma
RHOA: Transforming protein RhoA
HAT1: Histone acetyltransferase 1
BMP4: Bone morphogenetic proteins 4
HK1/2: Hexokinase 1/2
PFKFB3: 6-Phosphofructo-2-kinase/fructose-2, 6-bisphosphatase 3
PKM: Pyruvate Kinase M
G6PDH: Glucose-6-phosphate dehydrogenase
PGD: Phosphogluconate Dehydrogenase
TKT: Transketolase
KLF15: Kruppel Like Factor 15
AMBRA1: BECN1-regulated autophagy protein 1
LC3B: Microtubule-associated proteins 1A/1B light chain 3B
LAMP1: Lysosomal-associated membrane protein 1
RAB7: Ras-related protein 7
TFEB: Transcription factor EB
OXPHOS: Oxidative phosphorylation
