# Supplementary material for: Priming mesenchymal stem cells with α-synuclein enhances neuroprotective properties through induction of autophagy in Parkinsonian models
Source: Stem Cell Res Ther. 2022 Sep 24;13:483. doi: 10.1186/s13287-022-03139-w (PMC9509608; doi:10.1186/s13287-022-03139-w)
Supplement: Supplementary file 1 — Additional file 1. S1 table. RT-qPCR prime sequence. Forward and reverse primer sequences were designed using Prime-BLAST. [file 13287_2022_3139_MOESM1_ESM.docx]

**S1 table. RT-qPCR prime sequence**. Forward and reverse primer sequences were designed using Prime-BLAST

| *Gene Name* | sequence (5`-3`) | | Annealinig Temp.(℃) |
| --- | --- | --- | --- |
| *p16* | Forward Primer: | TCCTGATTGGCGGATAGAGC | 65 |
|  | Reverse Primer: | CCCTCGTCGAAAGTCTTCCA |  |
| *p21* | Forward Primer: | TGGAGACTCTCAGGGTCGAAA |  |
|  | Reverse Primer: | GGGCGA GAAGGCAAAATCTG |  |
| *NANOG* | Forward Primer: | CCCTCCTCCCATCCCTCATAG |  |
|  | Reverse Primer: | CCTCGCTGATTAGGCTCCA |  |
| *SOX2* | Forward Primer: | GAAGGATAAGTACACGCTGCC |  |
|  | Reverse Primer: | TAACTGTCCATGCGCTGGTTC |  |
| *PPARG* | Forward Primer: | ACTTTGGGATCAGCTCCGTG |  |
|  | Reverse Primer: | GGAGATGCAGGCTCCACTTT |  |
| *ROHA* | Forward Primer: | GTCCACGGTCTG GTCTTCAG |  |
|  | Reverse Primer: | TTTCCACAGGCTCCATCACC |  |
| *SMURF2* | Forward Primer: | CAAGCTGCGCCTGACAGTA |  |
|  | Reverse Primer: | GCGGTTGATGGCATTGGAAA |  |
| *HAT1* | Forward Primer: | GGAAATGGCGGGATTTGGTG |  |
|  | Reverse Primer: | TGTTGACAGGCTACCAGCAAT |  |
| *BMP4* | Forward Primer: | CGTCCAAGCTATCTCGAGCC |  |
|  | Reverse Primer: | ACGACCATCAGCATTCGGTT |  |
| *HK1* | Forward Primer: | CCCTGAGGACATCATGCGAG |  |
|  | Reverse Primer: | GATGGCCTTCCGGATCAGAG |  |
| *HK2* | Forward Primer: | CCCTGAGGACATCATGCGAG |  |
|  | Reverse Primer: | GATGGCCTTCCGGATCAGAG |  |
| *PFKFB3* | Forward Primer: | TGCAACTCTGCCTTATCC |  |
|  | Reverse Primer: | ATACACCTGCACAGCGTACAG |  |
| *G6PD* | Forward Primer: | TCCCGCATCTCTTTCACTCAC |  |
|  | Reverse Primer: | AGGAGAGGTGGTGGCGAGTAG |  |
| *PGD* | Forward Primer: | GTCCCACCCTTCCACTTT |  |
|  | Reverse Primer: | CCATCCTGTAGGCACTCC |  |
| *TKT* | Forward Primer: | CCTGGATGGGGACACCAAAA |  |
|  | Reverse Primer: | GCGTGAAGAAGGCTGCAAAA |  |
| *PKM* | Forward Primer: | AGGGAATCACGCCACTGC |  |
|  | Reverse Primer: | AGCCAGAGAAACCAGCCAAG |  |
| *LDH* | Forward Primer: | TGCTCAGCTCCCAGGTCAC |  |
|  | Reverse Primer: | GCCTTCAACTCCTTCATGGTCT |  |
| *GAPDH* | Forward Primer: | GGTGATGGCATGGACTGTGGT |  |
|  | Reverse Primer: | AAGGGTCATCATCTCTGCCC |  |
