# Supplementary material for: Priming mesenchymal stem cells with α-synuclein enhances neuroprotective properties through induction of autophagy in Parkinsonian models
Source: Stem Cell Res Ther. 2022 Sep 24;13:483. doi: 10.1186/s13287-022-03139-w (PMC9509608; doi:10.1186/s13287-022-03139-w)
Supplement: Supplementary file 2 — Additional file 2. Fig. S1. A characterization of fibrillary form of α-synuclein using the transmission electron microscopy. Fig. S2. A karyotype analysis of primed MSCs. Primed MSCs with α-synuclein had a normal karyotype with a diploid chromosome number as naïve MSCs in passage 8. [file 13287_2022_3139_MOESM2_ESM.docx]

SUPPLEMENTARY INFORMATION

**Priming mesenchymal stem cells with α-synuclein enhances neuroprotective properties through induction of autophagy in Parkinsonian models**

Jin Young Shin,^1,2^ Dong-Yeol Kim,^1,2^ Jieun Lee,^1,2^ Yu Jin Shin, ^1,2^ Phil Hyu Lee^1,2^

^1^Department of Neurology, Yonsei University College of Medicine, Seoul, South Korea

^2^Severance Biomedical Science Institute, Yonsei University College of Medicine, Seoul, South Korea

**Supplementary Figure 1**


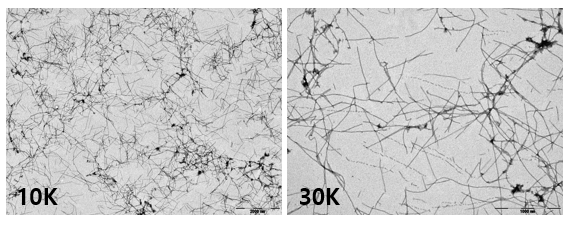


**Fig. S1.** A characterization of fibrillary form of α-synuclein using the transmission electron microscopy

**Supplementary Figure 2**


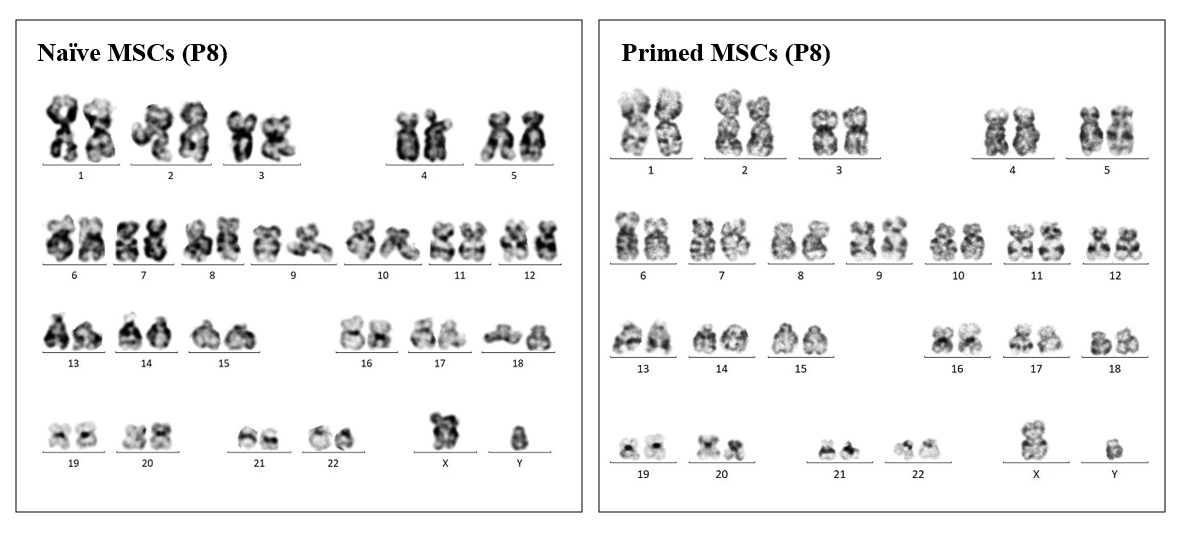


**Fig. S2**. **A karyotype analysis of primed MSCs.** Primed MSCs with α-synuclein had a normal karyotype with a diploid chromosome number as naïve MSCs in passage 8.
